# Virtual Care and Telehealth for Improving Healthcare Access in Rural Western Canada and the Western United States: A Scoping Review and Narrative Synthesis

**DOI:** 10.3390/jcm15124749

**Published:** 2026-06-18

**Authors:** Tomasz Karczewski, Jennifer M. L. Stephens, Dawid Karczewski, Sahar Feizizadeh, Avni K. Patel, Merjorie M. A. Pinero, Mihaela Olsen, Melanie L. Thompson

**Affiliations:** 1 Cranston Ridge Medical Clinic, Calgary, AB T3M 3A9, Canada; tomasz@cranstonridgemedical.com (T.K.); avni@cranstonridgemedical.com (A.K.P.); merjorie@cranstonridgemedical.com (M.M.A.P.); mihaela@cranstonridgemedical.com (M.O.); melanie@cranstonridgemedical.com (M.L.T.); 2 Fay W. Whitney School of Nursing, University of Wyoming, Laramie, WY 82071, USA; jsteph35@uwyo.edu; 3 Cranston Smart Drug Mart, Calgary, AB T3M 3A9, Canada; sahar@cranstonridgemedical.com

**Keywords:** telehealth, virtual care, rural health, healthcare access, western Canada, western United States, frontier health, Indigenous health, eConsult, narrative synthesis, scoping review

## Abstract

**Background/Objectives:** Western Canadian and U.S. communities outside urban centres remain underserved by primary, specialist, emergency, mental health, and chronic-disease services. These access problems reflect distance, weather, workforce shortages, specialist maldistribution, primary care attachment gaps, broadband limitations, and the governance realities of Indigenous and Tribal communities. This scoping review with narrative synthesis examined how telehealth and virtual-care models affect rural access in western Canada and the western/frontier United States. **Methods:** Searches were completed on 21 May 2026 in PubMed/MEDLINE, Embase, CINAHL, Scopus, the Cochrane Library, and PubMed Central. Supplementary searches included Google Scholar, publisher platforms, reference-list checking, and official Canadian and U.S. health-system sources. Peer-reviewed evidence published from 1 January 2016 to 21 May 2026 was eligible when it addressed rural, remote, frontier, Indigenous, underserved, western, or northern healthcare settings and reported access, implementation, safety, continuity, equity, or service-use outcomes. **Results:** The search identified 112 records; 27 duplicates were removed, 85 records were screened, 37 full texts were assessed, and 28 peer-reviewed records were included. Seven official sources were retained separately. Evidence was mainly observational, qualitative, mixed-methods, implementation-focused, or review-level. Moderate confidence supported telehealth for travel reduction and specialist input, especially through eConsultation, provider-to-provider consultation, telementoring, and real-time emergency support. Confidence was low to moderate for hybrid primary care and telemental health, and low for durable reductions in emergency department use. **Conclusions:** Telehealth may be most appropriately implemented as a hybrid, locally anchored, culturally safe access model, not as a stand-alone substitute for rural primary care, specialist capacity, or emergency services. Implementation should include broadband support, local physical assessment capacity, documentation, continuity, patient education, and clear escalation pathways.

## 1. Introduction

Access to health care is not simply the presence of a clinic, a professional title, or a scheduled appointment. It reflects the relationship between the availability, accommodation, affordability, acceptability, and appropriateness of services and the ability of patients and communities to perceive need, seek care, reach care, pay for care, and engage with care [1]. For rural and frontier communities, these dimensions often fail at several points at once: distance may delay care, weather may interrupt travel, local diagnostic capacity may be episodic, and small workforces may leave little redundancy when a clinician, clinic, or hospital unit becomes unavailable [2,3].

Western Canada and the western United States were examined together because the regions share important access mechanisms despite substantial policy differences. Both include large geographic areas, dispersed settlements, mountain, prairie, coastal or northern terrain, rural hospital vulnerability, workforce shortages, broadband gaps, and communities in which local relationships are central to care delivery [2,3,4,5,6,7]. At the same time, transferability cannot be assumed. Canada and the United States differ in healthcare financing, licensure, reimbursement, broadband policy, malpractice context, and the governance structures through which rural, Indigenous, and Tribal communities interact with health systems. This review therefore treats western Canada and the western United States as comparable rural access contexts, not interchangeable systems.

Telehealth and virtual care are often presented as solutions to rural geography, but the term covers several distinct service models. In this review, telehealth is used broadly to include patient-facing video or telephone visits, secure messaging, asynchronous communication, eConsultation, referral triage, remote monitoring, provider-to-provider virtual consultation, telementoring, tele-emergency support, and hybrid models in which virtual assessment is paired with local in-person backup [8,9,10]. These modalities differ clinically. A telephone follow-up for medication titration, a real-time rural emergency consultation, and an eConsult between a family physician and a specialist should not be interpreted as one intervention.

The access outcomes assessed in this review were mapped explicitly to rural service needs: timeliness of care, travel avoided, primary care attachment, specialist input, emergency department or urgent-care use, continuity, patient experience, clinician support, diagnostic and medication safety, digital equity, and cultural safety [1,2,3,8,9,10]. This broad access definition is consistent with rural health services research because the practical question is not whether a virtual visit occurred, but whether care became more timely, safer, more continuous, more appropriate, and more equitable.

Implementation conditions are central. Broadband access is now widely treated as a health-enabling infrastructure issue rather than a consumer convenience [4,5,7], and Canadian virtual-care use varies by sociodemographic and health-related factors [6]. Implementation frameworks such as NASSS and CFIR further emphasize that technology adoption depends on the condition being treated, user capability, workflow, organizational support, financing, policy, and sustainability rather than on the technology alone [11,12]. For Indigenous and Tribal communities, virtual care must also be understood through cultural safety, community governance, trust, privacy, language, and relationship-based care [13,14,15,16].

Three research questions guided the review: (1) Which telehealth and virtual-care models have been described or evaluated for rural, remote, frontier, Indigenous, or underserved settings relevant to western Canada and the western United States? (2) Which dimensions of access are most often addressed, including travel burden, timeliness, specialist input, emergency support, continuity, clinician support, safety, digital equity, and cultural safety? (3) Under what implementation conditions does the evidence suggest that virtual care extends local services rather than replacing them?

The purpose of this scoping review and narrative synthesis was to map and synthesize evidence on telehealth and virtual-care models relevant to healthcare access in rural western Canada and the western/frontier United States, with explicit attention to which evidence is directly applicable, which is mechanistically transferable, and where conclusions remain uncertain.

## 2. Materials and Methods

### 2.1. Review Design, Protocol, and Reporting Standard

A scoping review design was selected because the review question concerned the range, mechanisms, implementation conditions, and transferability of virtual-care models rather than a single intervention-outcome pair suitable for meta-analysis. The revised design is therefore best described as a scoping review with systematic searching and narrative synthesis. Reporting followed the PRISMA extension for scoping reviews (PRISMA-ScR) [17], consistent with established scoping-review methodological guidance [18], while PRISMA 2020 principles were used for the flow diagram and transparent search reporting [19].

A structured protocol was prepared before synthesis and is supplied as Supplementary File S2. The protocol specified the review question, eligibility criteria, information sources, search concepts, screening process, data items, critical-appraisal approach, transferability rules, and synthesis domains. The review was not registered in PROSPERO or another public registry. No amendments were made to the review question after protocol development; the manuscript terminology was revised to describe the design more accurately as a scoping review rather than a conventional systematic review of intervention effectiveness.

### 2.2. Review Question

The review question was: In rural, remote, frontier, Indigenous, and underserved communities in western Canada and the western United States, how do telehealth and virtual-care models influence access to healthcare services, including timeliness, travel burden, primary care attachment, specialist input, emergency department or urgent-care use, continuity, patient experience, clinician support, safety, digital equity, and cultural safety?

### 2.3. Eligibility Criteria and Transferability Rules

Eligibility criteria were specified using a population-concept-context structure. Direct evidence was defined as evidence from western Canadian provinces or territories, or western/frontier U.S. states, addressing rural, remote, Indigenous, frontier, or underserved settings. Mechanistically transferable evidence was retained when it came from another Canadian or U.S. rural, northern, remote, Indigenous, or underserved context and addressed an access mechanism directly relevant to the western Canada–western U.S. comparison, such as travel reduction, rural clinician support, eConsultation, hybrid primary care, or digital exclusion. Transferable evidence was not used to make jurisdiction-specific claims unless the implementation context was similar enough to justify cautious inference. The eligibility criteria and transferability rules used to select and interpret sources of evidence are summarized in Table 1.

### 2.4. Information Sources and Search Strategy

The final search date was 21 May 2026. Electronic database searches included PubMed/MEDLINE, Embase, CINAHL, Scopus, the Cochrane Library, and PubMed Central. Supplementary and institutional searches included Google Scholar, publisher platforms, reference lists of included reviews and primary studies, and official Canadian and U.S. sources relevant to broadband, shortage-area, telehealth, and rural health-system context. Official contextual sources included, among others, Innovation, Science and Economic Development Canada, the Canadian Radio-television and Telecommunications Commission, Statistics Canada, the Health Resources and Services Administration, the Agency for Healthcare Research and Quality, and the Rural Health Information Hub [2,3,4,5,6].

Core peer-reviewed searches covered 1 January 2016 through 21 May 2026. Earlier landmark conceptual or implementation sources were retained only when directly necessary for access theory, broadband-as-infrastructure framing, implementation-science interpretation, or scoping-review reporting [1,7,11,12,17,18,19]. Searches were limited to English-language records because reliable full-text interpretation in other languages was not available within the review process.

The core search concepts combined telehealth/telemedicine/virtual care terms with rural/remote/frontier/Indigenous/underserved terms, jurisdictional terms for western Canada and the western United States, and access outcomes. The PubMed/MEDLINE core string was: (telehealth[tiab] OR telemedicine[tiab] OR “virtual care”[tiab] OR “remote consultation”[tiab] OR eConsult[tiab] OR “remote monitoring”[tiab] OR “provider-to-provider”[tiab] OR “Project ECHO”[tiab]) AND (rural[tiab] OR remote[tiab] OR frontier[tiab] OR northern[tiab] OR Indigenous[tiab] OR underserved[tiab]) AND (Canada[tiab] OR Alberta[tiab] OR “British Columbia”[tiab] OR Saskatchewan[tiab] OR Manitoba[tiab] OR Yukon[tiab] OR “Northwest Territories”[tiab] OR “United States”[tiab] OR Wyoming[tiab] OR Montana[tiab] OR Idaho[tiab] OR Nevada[tiab] OR Utah[tiab] OR Arizona[tiab] OR “New Mexico”[tiab] OR Colorado[tiab] OR Washington[tiab] OR Oregon[tiab] OR California[tiab]) AND (access[tiab] OR timeliness[tiab] OR travel[tiab] OR “primary care”[tiab] OR specialist[tiab] OR “emergency department”[tiab] OR continuity[tiab] OR equity[tiab] OR broadband[tiab]) AND (“1 January 2016”[Date—Publication]: “21 May 2026”[Date—Publication]). Full database-specific strings are provided in Supplementary File S2. The information sources, final search date, and search approach used for each source are summarized in Table 2.

### 2.5. Selection of Sources and Data Charting

Records were de-duplicated and screened in two stages: title/abstract or search-snippet screening followed by full-text assessment. Database records with bibliographic metadata were screened by two reviewers against the pre-specified eligibility criteria. Records identified through publisher platforms, reference chasing, Google Scholar, and official institutional websites were first screened for relevance and then verified by at least one additional team member when selected for inclusion or when eligibility was uncertain. Full-text eligibility decisions were resolved by consensus.

Data were charted using a structured form. Extracted items included author and year, design, sample size or number of included studies, country/province/state, rurality definition, population, telehealth modality, comparator or context, follow-up period, access outcomes, clinical or service-use outcomes, equity and cultural-safety considerations, implementation barriers/facilitators, limitations, funding where reported, and applicability to western Canada or the western/frontier United States. To reduce selection and interpretive bias, the literature was scanned and then reviewed by a multidisciplinary team comprising advanced practice nurses, physicians, and pharmacists. Discrepancies or uncertainties about eligibility, extraction, or interpretation were resolved through team discussion. No automated eligibility decisions were used.

### 2.6. Critical Appraisal Methods

Critical appraisal was performed at the study level using CASP-informed criteria appropriate to the design: clarity of aims, appropriateness of design, sampling or data source, outcome ascertainment, confounding, reflexivity for qualitative studies, completeness of reporting, and applicability to the review question [20]. Review-level evidence was appraised for search transparency, eligibility clarity, synthesis approach, risk-of-bias assessment, and consistency between evidence and conclusions.

Certainty was summarized by outcome domain using GRADE concepts—risk of bias, inconsistency, indirectness, imprecision, and publication/reporting concerns—while recognizing that many sources were qualitative, mixed-methods, or implementation studies rather than trials [21]. Qualitative and mixed-methods findings were interpreted using CERQual-informed considerations, especially methodological limitations, coherence, adequacy, and relevance. Certainty ratings therefore represent confidence in a synthesized access mechanism, not a pooled causal effect.

### 2.7. Synthesis Methods

Narrative synthesis was selected because included sources differed substantially in geography, modality, population, comparator, outcome definition, follow-up, and policy context. Meta-analysis was not performed. Sources were grouped by access mechanism and evidence type: (1) rural access barriers and infrastructure; (2) patient-facing virtual care and uptake; (3) hybrid virtual/in-person primary care; (4) provider-to-provider telehealth, eConsultation, and telementoring; (5) emergency, specialty, chronic disease, and mental health applications; and (6) equity, cultural safety, and digital exclusion.

The synthesis distinguished direct evidence from mechanistically transferable evidence. Direct evidence received greater weight for jurisdiction-specific interpretation. Transferable evidence was used to support mechanisms and implementation logic only when the rural access problem and virtual-care model were sufficiently similar.

### 2.8. Reporting Bias Assessment

Formal funnel-plot or small-study-effect assessment was not appropriate because no meta-analysis was performed and the included sources were heterogeneous. Risk of missing evidence was addressed through searches across multiple bibliographic databases, reference-list checking, publisher-platform searches, Google Scholar, and official institutional sources. Reporting bias remains possible, particularly because successful virtual-care programs may be more likely to be described or published than unsuccessful or abandoned programs.

## 3. Results

### 3.1. Selection of Sources

**Figure 1:**
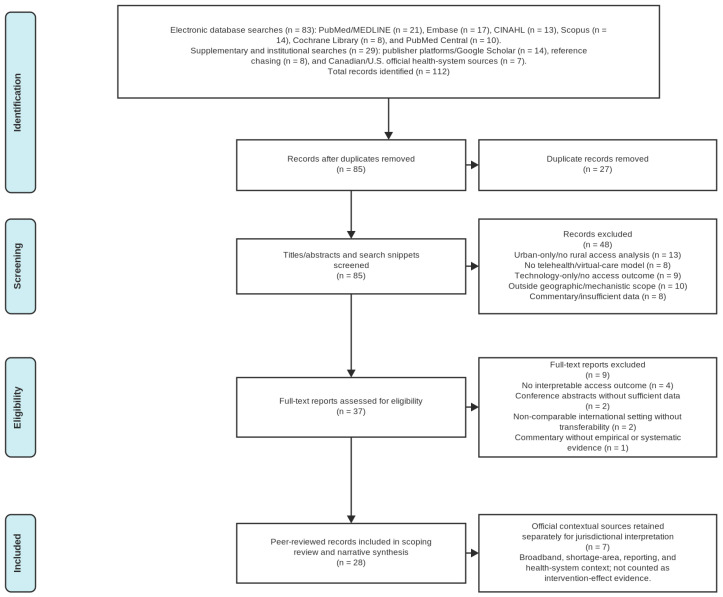
PRISMA-ScR-style flow diagram for identification, screening, eligibility assessment, and inclusion in the scoping review.

The search identified 112 records. After removal of 27 duplicates, 85 records were screened. Forty-eight were excluded at title/abstract or search-snippet stage because they were urban-only, technology-only, disease-efficacy studies without access outcomes, non-health digital-access sources, outside the geographic or mechanistic scope, or commentaries without empirical or systematic evidence. Thirty-seven full-text reports were assessed for eligibility. Nine were excluded: four lacked interpretable access outcomes, two were conference abstracts without sufficient data, two focused on non-comparable international settings without transferable content, and one was a commentary without empirical or systematic evidence. Twenty-eight peer-reviewed records were included in the scoping evidence synthesis. Seven official contextual sources were retained separately for broadband, shortage-area, reporting, and health-system interpretation. The study-selection process and reasons for exclusion at each stage are summarized in Figure 1. The corresponding numerical flow summary is provided in Table 3.

### 3.2. Characteristics of Included Evidence

The evidence base consisted mainly of observational, cross-sectional, qualitative, mixed-methods, implementation, and review-level studies. Randomized evidence was limited because rural virtual-care models are typically deployed as service pathways rather than patient-level randomized interventions. The included evidence addressed patient-facing telemedicine, rural telehealth uptake, hybrid primary care, eConsultation, provider-to-provider consultation, telementoring, telemental health, rural emergency support, remote monitoring, patient satisfaction, Indigenous virtual care, broadband, and digital equity.

Evidence was geographically uneven. Direct western Canadian evidence was strongest for British Columbia rural micropractice and Real-Time Virtual Support models. Direct western U.S. evidence was strongest for Wyoming Medicaid telehealth usability and broader U.S. frontier provider-to-provider telehealth evidence. Several Ontario and national Canadian studies were retained because they addressed mechanisms—rural uptake, hybrid primary care, eConsultation, attachment, and specialist access—that are clinically transferable to western rural settings, but these studies were interpreted cautiously because reimbursement, geography, and delivery-system context differ. The study-level evidence map for the key included peer-reviewed records is presented in Table 4.

### 3.3. Rural Access Barriers and Infrastructure Context

The contextual evidence reinforced that rural telehealth is not only a clinical modality; it is also an infrastructure intervention. Broadband policy in Canada treats high-speed connectivity as a condition for access to services, including health care [4,5]. U.S. shortage-area data similarly show persistent primary care and mental health workforce gaps in rural and frontier regions [3]. Broadband access, device access, privacy, digital literacy, reimbursement, workflow, and trust therefore determine whether telehealth narrows or widens rural access gaps.

The included studies consistently suggest that telehealth is most useful when it addresses a concrete rural access failure: travel burden, delayed specialist advice, lack of local clinician support, limited attachment, mental health access, or follow-up for stable chronic disease. Evidence did not support the assumption that rurality alone guarantees high telehealth uptake. In Ontario, telemedicine use increased sharply during the pandemic, but the increase was greater in urban and less-rural populations than in more-rural populations [22]. This finding is highly relevant to western Canada and the western United States because the communities with the greatest need may have the least digital readiness.

### 3.4. Patient-Facing Virtual Care and Rural Uptake

Patient-facing virtual care was most consistently associated with improved access when it displaced travel-intensive or routine follow-up care, such as medication review, chronic disease monitoring, mental health follow-up, rehabilitation, or preliminary triage. Rural U.S. review evidence and rural patient-satisfaction evidence support convenience, reduced travel, and favourable patient experience, but most studies were heterogeneous and not designed to establish durable outcome effects [8,35].

The Canadian rural uptake evidence is more cautious. Chu et al. found that telemedicine adoption increased in rural Ontario during the COVID-19 pandemic, but use increased more in urban and less-rural populations [22]. This finding supports an equity warning: telehealth expansion may preferentially benefit patients who already have the devices, bandwidth, privacy, and literacy needed to use it. Telephone access may therefore remain necessary in low-bandwidth or low-income rural settings rather than being treated as an inferior modality by default.

### 3.5. Hybrid Virtual/In-Person Rural Primary Care

The available evidence most consistently suggests that patient-facing rural primary care is safest and most acceptable when virtual access is paired with local in-person capacity. A British Columbia micropractice study found that patients and providers valued flexible asynchronous and virtual communication, but the physician identified concerns about missed diagnoses and coordination with specialists [24]. The study’s small sample limits generalizability, but it illustrates the central implementation issue: virtual access depends on trust, relationship continuity, and a clear plan for problems that require examination.

Ontario’s VTAC and Integrated Virtual Care studies provide a more developed hybrid model. These studies combined remote family-physician assessment with local in-person support, including community paramedics, clinical assessment centres, and attachment pathways for patients without a family physician [27,28,29,30,31]. Findings suggest improved patient experience, physician satisfaction, access for unattached patients, and reductions in low-acuity emergency department use in the evaluated setting [27,28,29,30,31]. Because these studies were conducted outside western Canada and were influenced by pandemic-era policy conditions, they are best interpreted as mechanistically transferable rather than directly generalizable.

### 3.6. Provider-to-Provider Telehealth, eConsultation, and Telementoring

Provider-to-provider telehealth was the most methodologically developed evidence area. The AHRQ comparative effectiveness review and related systematic review evaluated rural provider-to-provider telehealth across a large body of evidence and concluded that telehealth-supported collaboration may produce similar or better outcomes in selected domains, while also improving rural clinician knowledge, confidence, and self-efficacy [9,10]. Strength of evidence remained low in many clinical domains because studies were often observational, small, or vulnerable to time-trend bias, but the mechanism is directly relevant: telehealth supports the clinician who is physically present with the patient.

eConsultation and telementoring also address specialist maldistribution. eConsult studies show that asynchronous specialist advice can reduce avoidable face-to-face referral and provide timely input for remote or rural communities [38,39]. Project ECHO-style telementoring can increase provider knowledge and confidence, although evidence for patient outcomes is less robust and often based on observational or self-reported measures [40,41]. These models are especially relevant to western rural systems because they strengthen local care relationships rather than replacing them with parallel virtual-only services.

### 3.7. Emergency, Specialty, Chronic Disease, and Mental Health Applications

Emergency and urgent-care evidence was promising but less certain. British Columbia Real-Time Virtual Support is a directly relevant western Canadian example because it offers real-time virtual advice for rural, remote, First Nations, and emergency-care contexts [32,33,34]. Such models may reduce professional isolation, improve confidence, and help determine whether transfer is necessary. However, the evidence should not be interpreted as proving that telehealth reduces emergency department use in all settings. Appropriate virtual assessment may increase emergency referral or transfer when serious illness is identified.

Specialty and chronic disease applications were generally supportive but heterogeneous. Northern Canadian telehealth evidence supports reduced travel and improved specialist access in remote communities [23]. Rural cardiovascular and home-based digital-health reviews suggest potential benefits for follow-up, self-management, patient empowerment, and travel reduction, while also identifying infrastructure, workflow, and sustainability challenges [25,26]. Telemental health appears particularly suitable for rural access when privacy, crisis response, and continuity are maintained; a systematic review identified six eligible randomized studies and reported symptom improvements across several mental health conditions, though broader certainty was limited [36].

### 3.8. Equity, Cultural Safety, and Digital Exclusion

Equity findings were consistent across contexts: telehealth can reduce travel, cost, and missed work, but it is not equitable by default. Wyoming Medicaid evidence supports telehealth usability in a rural and low-income population, while also demonstrating the importance of usability, visit type, rurality, and digital access [37]. These findings are directly relevant to western U.S. frontier settings, where distance and poverty may intersect with broadband and device limitations.

Indigenous-focused evidence strongly indicates that virtual care must be co-designed, culturally safe, relationship-centred, and governed with Indigenous communities [13,14,15,16]. Rapid and scoping reviews emphasize technology access, cultural safety, therapeutic relationships, privacy, and community involvement [13,14]. Alberta-based ARQS work further shows that Indigenous virtual primary care quality depends on access, relationships, quality, and safety, and that patient-experience tools should be grounded in Indigenous perspectives [15,16]. These findings limit any conclusion that virtual care alone can solve rural Indigenous access inequity.

### 3.9. Critical Appraisal and Certainty of Evidence

Study-level limitations were common. Observational and service-evaluation studies were often vulnerable to pandemic-era confounding, selection bias, incomplete adjustment for secular trends, self-selected patient-experience samples, and limited follow-up. Qualitative studies provided useful implementation and patient-experience detail but were frequently small and context-specific. Review-level evidence was stronger where searches, dual screening, and structured appraisal were reported, as in the AHRQ provider-to-provider telehealth review [10].

Overall certainty was highest for the direction of effect that telehealth can reduce travel and enable specialist input when infrastructure and workflow support exist. Certainty was moderate for provider-to-provider support because the evidence base was broader and more systematically assessed. Certainty was lower for emergency department utilization and for direct transferability between western Canada and the western United States. Certainty that telehealth is equitable by default was very low; the evidence instead indicates that equity depends on design and implementation. The CASP-informed and GRADE/CERQual-informed certainty summary by synthesis domain is presented in Table 5.

## 4. Discussion

### 4.1. Principal Interpretation

The central finding is deliberately modest: telehealth may extend rural healthcare access when it is implemented as part of a local care system. The strongest and most coherent evidence supports virtual care as a means of reducing avoidable travel, improving access to specialist input, supporting rural clinicians, and maintaining follow-up for selected chronic disease, rehabilitation, and mental health needs. The evidence is weaker for virtual-only replacement models and for durable reductions in emergency department use.

This distinction matters. A virtual visit can be clinically useful, but a virtual network that links patients, local clinicians, community paramedics, nurses, pharmacists, Indigenous health workers, rural hospitals, and specialists is more likely to improve access without undermining continuity. The evidence therefore supports an implementation recommendation—not a proven causal hierarchy—that rural telehealth should be virtual-enabled and locally anchored rather than virtual-only.

### 4.2. Western Canada and Western U.S. Transferability

The western Canada–western U.S. comparison is useful because the two regions share rural access mechanisms, but it must be interpreted with caution. Similarities include distance, weather, workforce shortage, specialist maldistribution, frontier service configurations, Indigenous and Tribal community contexts, and broadband gaps [2,3,4,5,6,7,13,14,15,16]. Differences include payment, licensure, malpractice, scope of practice, Medicaid and provincial reimbursement rules, and the legal and governance relationships between health systems and Indigenous or Tribal authorities. These differences mean that access mechanisms may transfer more readily than exact effect sizes or implementation models.

For western Canada, the most directly applicable evidence comes from northern Canadian telehealth [23], rural British Columbia primary care [24], British Columbia Real-Time Virtual Support [32,33,34], and Canadian broadband policy [4,5]. For the western United States, Wyoming Medicaid usability evidence is directly relevant to rural and low-income telehealth access [37], while U.S. provider-to-provider telehealth evidence is relevant to frontier clinician support [9,10]. Ontario hybrid primary-care and eConsult evidence was retained because it illuminates transferable mechanisms—hybrid local backup, attachment pathways, referral avoidance, and specialist advice—but it should not be read as direct proof that the same effect size would occur in Alberta, British Columbia, Saskatchewan, Manitoba, Wyoming, Montana, Idaho, or other frontier settings [27,28,29,30,31,38,39].

### 4.3. Evidence Versus Policy Interpretation

Several recommendations in rural virtual care are clinically plausible but not yet proven by direct comparative evidence. The phrase ‘virtual-first, not virtual-only’ is therefore better understood as an implementation principle than as a trial-proven conclusion. It follows from converging evidence on diagnostic limitations, patient preference, rural clinician support, hybrid primary care, and safety escalation rather than from direct randomized comparisons of virtual-only versus hybrid rural systems [24,27,28,29,30,31,32,33,34].

Emergency department outcomes require similar caution. Virtual care may reduce low-acuity emergency department visits when patients receive timely alternative assessment, but appropriate escalation may increase emergency referral, transfer, or admission when serious illness is detected [27,28,32,33,34]. Reductions in emergency department volume should therefore never be the sole measure of success; diagnostic safety, appropriate transfer, patient outcomes, and continuity must be measured alongside utilization.

### 4.4. Communication, Patient Engagement, and Chronic Care

Successful rural telehealth is partly a communication intervention. Virtual modalities work best when they improve explanation, education, adherence support, follow-up, and coordination among patients, families, local clinicians, and specialists [8,9,10,27,28,29,30,31,38,39,40,41,42]. A contemporary review by Costa et al. focuses on chronic lower limb wounds rather than rural telehealth, but it reinforces a theme directly relevant to this review: chronic care depends on communication, patient education, health literacy, interdisciplinary coordination, and culturally appropriate engagement [42]. This supports the present review’s emphasis on local anchoring and patient-centred implementation, especially for chronic disease, wound care, medication review, and remote monitoring.

### 4.5. Implementation Model

A defensible rural telehealth model should begin with the access problem it is trying to solve. For a patient living far from a specialist, the appropriate model may be eConsultation, remote specialist advice, or provider-to-provider consultation. For an unattached patient, the model may require hybrid primary care with a named clinician, local assessment capacity, and attachment pathways. For a rural clinician managing acute illness, the model may require real-time emergency support, transfer protocols, and equipment readiness. For Indigenous and Tribal communities, the model must be co-designed with community governance, cultural safety, privacy, and language supports.

Evaluation should measure access and harms together. Core outcomes should include same- or next-day access, travel avoided, specialist wait time, primary care attachment, continuity, emergency department use, transfer rates, hospitalization, diagnostic safety, medication safety, patient-reported access, clinician confidence, digital exclusion, cultural safety, cost, and sustainability. Measuring virtual visit volume alone is inadequate and may reward activity without demonstrating better care. The proposed implementation components, functions, and safeguards for telehealth-enabled rural healthcare access are summarized in Table 6.

### 4.6. Strengths and Limitations

Strengths of this review include a defined question, explicit eligibility criteria, multiple bibliographic and institutional information sources, reconciled search dates, a PRISMA-ScR reporting approach, structured data charting, separation of peer-reviewed evidence from official contextual sources, CASP-informed critical appraisal, GRADE- and CERQual-informed certainty interpretation, and explicit distinction between direct and mechanistically transferable evidence. The multidisciplinary team of advanced practice nurses, physicians, and pharmacists strengthened interpretation across clinical, rural, medication, and implementation perspectives.

Limitations are important. The review was not prospectively registered in a public registry. Although a protocol is supplied as Supplementary Material, it was not publicly available before manuscript submission. Some evidence comes from Ontario, national Canadian sources, or broad Indigenous telehealth reviews rather than directly from western Canada or the western United States. Many studies were conducted during or after the COVID-19 expansion of virtual care, which creates confounding by temporary billing changes, emergency funding, infection-control needs, and altered patient behaviour. Several studies used self-reported experience outcomes, short follow-up, and non-randomized designs. Meta-analysis was not appropriate because models, outcomes, populations, and settings were too heterogeneous.

### 4.7. Research Implications

Future research should move from descriptive adoption studies to prospective implementation and effectiveness evaluation. Useful designs include stepped-wedge trials, interrupted time-series analyses, matched rural comparator studies, hybrid effectiveness-implementation trials, and linked administrative-data analyses. Studies should clearly define the virtual-care model, staffing, reimbursement, local physical backup, escalation rules, rurality, Indigenous or Tribal governance, digital supports, and continuity mechanisms.

The most useful comparative research for western Canada and the western United States would evaluate similar outcome sets across jurisdictions while respecting policy differences. This would allow assessment of what transfers across rural systems and what must be locally adapted. In particular, emergency, chronic disease, mental health, Indigenous virtual primary care, and provider-to-provider support require evaluations that measure both access gains and unintended harms.

## 5. Conclusions

Telehealth and virtual care may improve healthcare access in rural western Canada and the western United States when they are implemented with adequate broadband, local clinical backup, culturally safe design, patient education, documentation, continuity, and clear escalation pathways. The principal findings are:

- Telehealth appears most useful as an access extender for travel-intensive follow-up, specialist input, provider-to-provider support, eConsultation, telementoring, selected chronic disease and mental health follow-up, and rural emergency backup.
- The evidence is less certain for virtual-only models and for durable reductions in emergency department use, particularly where local examination capacity and escalation pathways are weak.
- Rural and frontier communities may benefit when virtual care reduces travel, supports local clinicians, expands specialist reach, and connects patients to ongoing care, but those benefits depend on implementation conditions.
- The impact of virtual care should be judged not by virtual visit volume alone, but by whether care becomes more timely, continuous, safe, equitable, culturally appropriate, and locally anchored.

## Figures and Tables

**Table 1:** Eligibility criteria and transferability rules for the scoping review.

Exclusion Criteria	Inclusion Criteria	Domain
Urban-only studies without rural analysis; inpatient-only studies without access relevance; pediatric-only studies unless the model applied to whole-community access.	Adults, families, clinicians, or whole-community populations in rural, remote, frontier, northern, Indigenous, underserved, western Canadian, or western/frontier U.S. settings. Mechanistically transferable Canadian or U.S. rural evidence retained when relevant.	Population
Technical platform papers without health access outcomes; electronic records without a virtual-care access component; interventions not connected to healthcare access.	Telehealth, telemedicine, virtual care, telephone/video care, secure messaging, eConsult, remote monitoring, provider-to-provider telehealth, tele-emergency support, Project ECHO/telementoring, or hybrid virtual/in-person care.	Concept/intervention
No interpretable access, implementation, service-use, patient-experience, safety, or equity outcome.	Usual care; pre-post implementation; rural versus urban use; virtual versus nonvirtual access; shortage-area, broadband, Indigenous/Tribal, or rural implementation context.	Context/comparator
Disease efficacy only without access or implementation relevance.	Timeliness, travel burden, attachment, specialist input, ED/urgent-care use, hospitalization, clinician confidence, patient satisfaction, digital equity, cultural safety, continuity, safety, implementation barriers/facilitators.	Outcomes
Editorials, opinion pieces, non-systematic commentaries, conference abstracts without sufficient data, non-English records not reliably interpretable.	Scoping/systematic/narrative reviews, randomized/quasi-experimental studies, cohort/cross-sectional studies, mixed-methods, qualitative studies, case studies, and official policy/context sources.	Designs
Superseded policy documents or sources not applicable to the review question.	Core peer-reviewed evidence from 1 January 2016 to 21 May 2026. Earlier landmark conceptual/reporting/implementation sources retained when directly necessary.	Publication window

**Table 2:** Information sources and search approach.

Search Approach	Date Searched	Source
Telehealth/telemedicine/virtual care/eConsult/remote monitoring/provider-to-provider/Project ECHO AND rural/remote/frontier/northern/Indigenous/underserved AND western Canada/western U.S. terms AND access outcomes; 2016–2026.	21 May 2026	PubMed/MEDLINE
Emtree and free-text terms adapted from PubMed: telehealth, telemedicine, remote consultation, virtual care, eConsult, rural health, Indigenous health, remote area, healthcare access, emergency department, continuity.	21 May 2026	Embase
CINAHL subject headings and free-text terms for telehealth, virtual care, rural health services, Indigenous populations, primary care, specialty access, emergency care, patient experience, equity.	21 May 2026	CINAHL
TITLE-ABS-KEY search combining telehealth/virtual-care terms with rural/frontier/Indigenous terms, jurisdictional terms, and access/service-use outcomes.	21 May 2026	Scopus
telehealth OR telemedicine OR virtual care OR eConsult OR remote monitoring AND rural OR remote OR frontier OR Indigenous, limited to relevant reviews and trials.	21 May 2026	Cochrane Library
Full-text verification and retrieval for indexed records; publisher searches for recently indexed or early online articles.	21 May 2026	PubMed Central/publisher platforms
Targeted citation chasing from included reviews and key primary studies; first relevant pages screened for exact combinations of rural telehealth, western Canada, western U.S., eConsult, and Indigenous virtual care.	21 May 2026	Google Scholar/reference lists
Canadian and U.S. health-system, broadband, shortage-area, rural health, and telehealth sources retained for contextual interpretation only unless original data were provided.	21 May 2026	Official/institutional sources

**Table 3:** Flow summary for the completed literature search.

n	Description	PRISMA Stage
112	Records identified through electronic database, supplementary, institutional, citation, and official-source searches	Identification
27	Duplicate records removed	De-duplication
85	Records screened	Screening
48	Records excluded at title/abstract/search-snippet stage	Screening exclusions
37	Full-text reports assessed for eligibility	Eligibility
9	Reports excluded with reasons	Full-text exclusions
28	Peer-reviewed records included in scoping synthesis	Included evidence
7	Official contextual sources retained separately	Contextual sources

**Table 4:** Study-level evidence map for key included peer-reviewed records.

Key Findings and Applicability	Modality/Outcomes	Setting and Rurality	Design/Sample	Source
Telemedicine increased sharply during COVID-19, but use increased more in urban and less-rural populations. Mechanistically transferable caution for western Canada: need does not equal uptake.	Telephone/video telemedicine uptake; rural vs. urban comparison; Jan 2012–Jun 2020.	Ontario; rurality based on Rurality Index for Ontario.	Population-based repeated cross-sectional administrative study; rural Ontario telemedicine users increased to 290,401 in 2020.	Chu et al., 2021 [22]
Reported generally positive outcomes and satisfaction, but heterogeneity and limited design rigour reduce certainty. Useful as broad U.S. rural context.	Patient and provider telehealth across chronic disease, specialty care, determinants of health.	U.S. rural communities; varied definitions.	Narrative review; 15 rural U.S. telehealth studies.	Butzner and Cuffee, 2021 [8]
Most robust evidence area. Findings suggest similar or improved outcomes in selected domains and better clinician knowledge/confidence, with low strength of evidence in many clinical areas.	Provider-to-provider consultation, tele-emergency, tele-ICU, education/mentoring; strength-of-evidence assessment.	Rural provider-to-provider telehealth; U.S. and broader rural settings.	Systematic/AHRQ review; 166 studies in 179 publications; 97 effectiveness studies.	Totten et al., 2024/AHRQ 2022 [9,10]
Directly relevant to northern and remote Canadian access mechanisms; supports travel reduction and specialist access, but program context limits generalizability.	Telehealth for specialist access and travel reduction.	Northern Canadian communities; remote and travel-intensive settings.	Northern/rural telehealth implementation evidence.	Jong et al., 2019 [23]
Patients and providers valued flexible communication and engagement; diagnostic limitations and specialist coordination remained concerns. Direct western Canadian evidence.	Patient portal, asynchronous communication, telephone/video hybrid care.	Rural British Columbia micropractice.	Qualitative study; 3 focus groups: 8 patients and 2 providers.	Burton et al., 2022 [24]
Potential for follow-up, self-management, and travel reduction; limited hard outcome evidence and implementation heterogeneity.	Digital virtual-care tools for cardiovascular follow-up and self-management.	Rural Canada.	Scoping review of rural Canadians with cardiovascular disease.	Buyting et al., 2022 [25]
Identified benefits and barriers related to infrastructure, literacy, workload, and sustainability. Useful for chronic-care implementation.	Home-based digital health, remote monitoring, virtual support.	Rural Canada.	Scoping review of home-based digital health in rural Canada.	Lai et al., 2026 [26]
Physicians reported positive clinical impact, satisfaction, and triage value; challenges included continuity and local resource navigation. Mechanistically transferable.	Virtual Triage and Assessment Centre; hybrid virtual/in-person care with community paramedics.	Renfrew County, rural Ontario.	Mixed-methods physician study; 17 survey respondents and 9 focus-group participants.	Fitzsimon et al., 2023 [27]
Renfrew County had larger declines in ED visits and hospitalizations than comparators; VTAC low-acuity ED visits decreased, but causal certainty limited by design and pandemic context.	VTAC hybrid model; ED visits, hospitalizations, costs.	Renfrew County and comparator rural regions.	Cross-sectional population-based comparative study; rural Ontario public health units, 2018–2021.	Fitzsimon et al., 2023 [28]
Demonstrated capacity to attach previously unattached patients. Transferable to western rural attachment gaps if local team infrastructure exists.	Hybrid attachment pathway to family physician and team-based primary care.	Rural Ontario Integrated Virtual Care model.	Program evaluation of new patient attachment.	Peixoto et al., 2024 [29]
Patient experience evidence supported access and satisfaction while identifying the need for clear navigation and escalation. Mechanistically transferable.	VTAC virtual, hybrid, and in-person modalities.	Renfrew County, rural Ontario.	Mixed-methods patient-experience study.	St-Amant et al., 2025 [30]
85% reported being satisfied or very satisfied; respondent selection and local context limit certainty.	Hybrid family physician/team-based primary care; satisfaction and access outcomes.	Rural Ontario Integrated Virtual Care.	Cross-sectional survey; 198/790 patients responded.	Buchanan et al., 2025 [31]
Direct western Canadian evidence supporting real-time rural clinician support and emergency advice; rigorous outcome evaluation remains limited.	Real-Time Virtual Support for rural clinicians and communities.	British Columbia rural, remote, First Nations, and emergency-care settings.	Descriptive case studies and health-system reports.	Lauscher et al.; Ho et al. [32,33,34]
Patients generally reported satisfaction; evidence base small and not specific to western Canada/U.S.	OT/PT/SLP telehealth satisfaction.	Rural rehabilitation settings.	Systematic review; 4 peer-reviewed rehabilitation studies.	Harkey et al., 2020 [35]
Reported improvements in symptoms across included studies; privacy, crisis pathways, and continuity remain necessary implementation safeguards.	Telemental health for mental disorders.	Rural mental health settings.	Systematic review; 6 randomized trials.	Watanabe et al., 2023 [36]
Direct western/frontier U.S. evidence. Supports usability but highlights the need to account for digital access and patient circumstances.	Telehealth usability; rurality, visit type, and demographics.	Wyoming; rural and low-income Medicaid population.	Survey of Wyoming Medicaid members.	Homer et al., 2024 [37]
Supports culturally safe, co-designed telehealth; directly relevant to First Nations and Tribal implementation principles.	Telehealth use, cultural safety, therapeutic relationships.	Australia, Canada, New Zealand, and the U.S.	Scoping review of Indigenous telehealth.	Moecke et al., 2024 [13]
High-quality Indigenous virtual care depends on access, relationships, quality, and safety; supports community-grounded evaluation.	Virtual primary care quality, cultural safety, relationships, patient experience.	Indigenous virtual primary care, including Alberta/Canadian contexts.	Rapid evidence review and qualitative ARQS studies.	Fitzpatrick et al., 2023; Roach et al., 2022/2023 [14,15,16]
eConsult can reduce specialist-access barriers; western-specific evidence indirect but mechanism highly transferable.	Asynchronous specialist advice; referral avoidance, response times, cost, rural use.	Nunavut and Ontario rural/urban settings.	Remote-community eConsult study and Ontario rural-urban eConsult analysis; 72,948 cases in 2021 in the 2025 study.	Liddy et al., 2017/2025 [38,39]
Improves provider knowledge/confidence in many studies; patient-outcome evidence weaker. Relevant for rural workforce support.	Telementoring and case-based specialty education.	Rural and underserved provider education contexts.	Systematic review of 52 ECHO studies plus official program context.	McBain et al., 2019/AHRQ Project ECHO [40,41]

**Table 5:** CASP-informed and GRADE/CERQual-informed certainty summary by synthesis domain.

Interpretation	Certainty/Confidence	Main Limitations	Contributing Evidence	Finding
Reasonable access benefit when virtual care replaces travel-intensive follow-up or specialist input.	Moderate	Travel avoided not consistently measured; models differ; patient selection likely.	Northern Canadian telehealth, rural U.S. reviews, rural patient-experience studies, eConsult evidence [8,23,24,25,26,35,38].	Telehealth can reduce travel burden in rural and remote settings.
Best interpreted as an implementation principle, not a proven causal superiority claim.	Low to moderate	Mostly observational/qualitative; Ontario evidence indirect for western Canada/U.S.; pandemic-era confounding.	BC micropractice and Ontario VTAC/IVC studies [24,27,28,29,30,31].	Hybrid virtual/in-person primary care may improve access more safely than virtual-only care.
Strongest evidence mechanism for rural access extension.	Moderate	Clinical domains vary; many studies low strength of evidence; patient outcomes inconsistent.	Totten/AHRQ systematic evidence, eConsult, Project ECHO [9,10,38,39,40,41].	Provider-to-provider telehealth improves rural clinician support and access to expertise.
Use ED volume cautiously; pair with safety and appropriateness outcomes.	Low	Nonrandomized studies; ED reductions may reflect pandemic trends; appropriate escalation may increase ED or transfer use.	VTAC studies, Real-Time Virtual Support, provider-to-provider reviews [27,28,32,33,34].	Telehealth reduces emergency department utilization.
Suitable domain for virtual access when local escalation and privacy are ensured.	Low to moderate	Small evidence base; crisis pathways and privacy vary.	Rural telemental health systematic review [36].	Telemental health improves rural access and symptoms.
Evidence points in the opposite direction: equity requires design, resources, and governance.	Very low	Equity barriers vary by community; broadband, device, literacy, cost, language, privacy, and trust not consistently measured.	Wyoming Medicaid usability, Indigenous and digital-equity evidence [13,14,15,16,37].	Telehealth is equitable by default.
Mechanisms transfer more readily than effect sizes or implementation models.	Low	Financing, licensure, reimbursement, Indigenous/Tribal governance, and infrastructure differ.	Regional and transferable rural studies [8,9,10,13,14,15,16,22,23,24,25,26,27,28,29,30,31,32,33,34,35,36,37,38,39,40,41].	Findings transfer directly between western Canada and the western U.S.

**Table 6:** Implementation model for telehealth-enabled rural healthcare access.

Required Safeguards	Function	Component
Red-flag criteria, same-day in-person backup, documentation to regular clinic, modality choice based on risk and patient preference.	Use telephone, video, or secure messaging for low-risk triage, follow-up, medication review, chronic disease monitoring, and mental health check-ins.	Access triage
Defined examination, specimen collection, point-of-care testing, wound care, immunization, and transfer pathways.	Connect virtual clinicians with rural nurses, paramedics, pharmacists, Indigenous health workers, clinics, or hospitals.	Local physical backup
Reliable technology, funding for clinician time, 24/7 or extended-hours coverage where needed, regional governance.	Offer real-time specialist or emergency advice to rural clinicians.	Provider-to-provider support
Response-time targets, EMR/referral integration, clear accountability for follow-up.	Use structured specialist advice for nonurgent clinical questions to reduce avoidable travel and referral.	eConsult and asynchronous advice
Device access, training, escalation thresholds, responsibility for reviewing incoming data.	Use home blood pressure, cardiac, respiratory, diabetes, wound, or palliative monitoring where clinically suitable.	Remote monitoring
Community governance, language support, privacy, telephone options, digital navigators, culturally safe care processes.	Co-design services with rural, Indigenous, Tribal, low-income, older adult, disabled, and language-minority users.	Equity and cultural safety
Pre-specified outcomes, rurality strata, patient-reported access, clinician experience, unintended harms, sustainability, and cost.	Measure access, continuity, safety, equity, and utilization rather than virtual visit volume alone.	Evaluation

## Data Availability

All data supporting this review are included in the manuscript and supplementary material. The search strategy, eligibility criteria, study-selection counts, included records, contextual sources, data-charting domains, and synthesis approach are reported in the manuscript and Supplementary File S2. Additional search-log details are available from the corresponding author upon reasonable request.

## Supplementary Materials

The following supporting information can be downloaded at: https://www.mdpi.com/article/10.3390/jcm15124749/s1, Supplementary File S1: Completed PRISMA-ScR checklist in the journal-provided template. Supplementary File S2: Search protocol, full database-specific search strings, data-charting template, and study-level critical appraisal matrix. The sources referenced in the Supplementary File S2 appraisal matrix are also cited in the main text and included in the reference list [8,9,10,13,14,15,16,22,23,24,25,26,27,28,29,30,31,32,33,34,35,36,37,38,39,40,41].

## Abbreviations

AHRQ	Agency for Healthcare Research and Quality
ARQS	Access, Relationships, Quality and Safety
CASP	Critical Appraisal Skills Programme
CERQual	Confidence in the Evidence from Reviews of Qualitative Research
CFIR	Consolidated Framework for Implementation Research
CINAHL	Cumulative Index to Nursing and Allied Health Literature
CRTC	Canadian Radio-television and Telecommunications Commission
ED	emergency department
GRADE	Grading of Recommendations Assessment, Development and Evaluation
HRSA	Health Resources and Services Administration
ISED	Innovation, Science and Economic Development Canada
NASSS	Nonadoption, Abandonment, Scale-up, Spread, and Sustainability
PRISMA	Preferred Reporting Items for Systematic Reviews and Meta-Analyses
PRISMA-ScR	Preferred Reporting Items for Systematic Reviews and Meta-Analyses extension for Scoping Reviews
U.S.	United States
VTAC	Virtual Triage and Assessment Centre
