# Supplementary material for: Virtual Care and Telehealth for Improving Healthcare Access in Rural Western Canada and the Western United States: A Scoping Review and Narrative Synthesis
Source: J Clin Med. 2026 Jun 18;15(12):4749. doi: 10.3390/jcm15124749 (PMC13302397; doi:10.3390/jcm15124749)
Supplement: Supplementary file 1 [file jcm-15-04749-s001.zip › jcm-4377842-supplementary.pdf]

Preferred Reporting Items for Systematic reviews and Meta-Analyses extension for Scoping Reviews (PRISMA-ScR) Checklist

SECTION	ITEM	PRISMA-ScR CHECKLIST ITEM	REPORTED ON PAGE #
**TITLE**			
Title	1	Identify the report as a scoping review.	p. 1
**ABSTRACT**			
Structured summary	2	Provide a structured summary that includes (as applicable): background, objectives, eligibility criteria, sources of evidence, charting methods, results, and conclusions that relate to the review questions and objectives.	pp. 1-2
**INTRODUCTION**			
Rationale	3	Describe the rationale for the review in the context of what is already known. Explain why the review questions/objectives lend themselves to a scoping review approach.	pp. 2-5
Objectives	4	Provide an explicit statement of the questions and objectives being addressed with reference to their key elements (e.g., population or participants, concepts, and context) or other relevant key elements used to conceptualize the review questions and/or objectives.	p. 5
**METHODS**			
Protocol and registration	5	Indicate whether a review protocol exists; state if and where it can be accessed (e.g., a Web address); and if available, provide registration information, including the registration number.	pp. 5-6; Suppl. S2
Eligibility criteria	6	Specify characteristics of the sources of evidence used as eligibility criteria (e.g., years considered, language, and publication status), and provide a rationale.	pp. 6-8; Table 1
Information sources*	7	Describe all information sources in the search (e.g., databases with dates of coverage and contact with authors to identify additional sources), as well as the date the most recent search was executed.	pp. 8-9; Table 2
Search	8	Present the full electronic search strategy for at least 1 database, including any limits used, such that it could be repeated.	pp. 8-9; Suppl. S2
Selection of sources of evidence†	9	State the process for selecting sources of evidence (i.e., screening and eligibility) included in the scoping review.	p. 9; pp. 10-11; Fig. 1; Table 3
Data charting process‡	10	Describe the methods of charting data from the included sources of evidence (e.g., calibrated forms or forms that have been tested by the team before their use, and whether data charting was done independently or in duplicate) and any processes for obtaining and confirming data from investigators.	p. 9; Suppl. S2
Data items	11	List and define all variables for which data were sought and any assumptions and simplifications made.	p. 9; Suppl. S2
Critical appraisal of individual sources of evidence§	12	If done, provide a rationale for conducting a critical appraisal of included sources of evidence; describe the methods used and how this information was used in any data synthesis (if appropriate).	pp. 9, 18-19; Table 5; Suppl. S2

SECTION	ITEM	PRISMA-ScR CHECKLIST ITEM	REPORTED ON PAGE #
Synthesis of results	13	Describe the methods of handling and summarizing the data that were charted.	p. 9
**RESULTS**			
Selection of sources of evidence	14	Give numbers of sources of evidence screened, assessed for eligibility, and included in the review, with reasons for exclusions at each stage, ideally using a flow diagram.	pp. 9-11; Fig. 1; Table 3
Characteristics of sources of evidence	15	For each source of evidence, present characteristics for which data were charted and provide the citations.	pp. 11-18; Table 4
Critical appraisal within sources of evidence	16	If done, present data on critical appraisal of included sources of evidence (see item 12).	pp. 18-19; Table 5; Suppl. S2
Results of individual sources of evidence	17	For each included source of evidence, present the relevant data that were charted that relate to the review questions and objectives.	pp. 11-19; Table 4; Results 3.3-3.8
Synthesis of results	18	Summarize and/or present the charting results as they relate to the review questions and objectives.	pp. 18-19; Table 5
**DISCUSSION**			
Summary of evidence	19	Summarize the main results (including an overview of concepts, themes, and types of evidence available), link to the review questions and objectives, and consider the relevance to key groups.	pp. 20-23; Table 6
Limitations	20	Discuss the limitations of the scoping review process.	p. 23
Conclusions	21	Provide a general interpretation of the results with respect to the review questions and objectives, as well as potential implications and/or next steps.	pp. 23-24
**FUNDING**			
Funding	22	Describe sources of funding for the included sources of evidence, as well as sources of funding for the scoping review. Describe the role of the funders of the scoping review.	p. 24

JBI = Joanna Briggs Institute; PRISMA-ScR = Preferred Reporting Items for Systematic reviews and Meta-Analyses extension for Scoping Reviews.

* Where *sources of evidence* (see second footnote) are compiled from, such as bibliographic databases, social media platforms, and Web sites.

† A more inclusive/heterogeneous term used to account for the different types of evidence or data sources (e.g., quantitative and/or qualitative research, expert opinion, and policy documents) that may be eligible in a scoping review as opposed to only studies. This is not to be confused with *information sources* (see first footnote).

‡ The frameworks by Arksey and O'Malley (6) and Levac and colleagues (7) and the JBI guidance (4, 5) refer to the process of data extraction in a scoping review as data charting.

§ The process of systematically examining research evidence to assess its validity, results, and relevance before using it to inform a decision. This term is used for items 12 and 19 instead of "risk of bias" (which is more applicable to systematic reviews of interventions) to include and acknowledge the various sources of evidence that may be used in a scoping review (e.g., quantitative and/or qualitative research, expert opinion, and policy document).

From: Tricco AC, Lillie E, Zarin W, O'Brien KK, Colquhoun H, Levac D, et al. PRISMA Extension for Scoping Reviews (PRISMAScR): Checklist and Explanation. Ann Intern Med. 2018;169:467–473. doi: 10.7326/M18-0850.

Supplementary File S2. Search Protocol, Search Strings, Data-Charting Form, and Critical Appraisal Matrix

This supplementary file provides detailed protocol elements and search information for the scoping review entitled: Virtual Care and Telehealth for Improving Healthcare Access in Rural Western Canada and the Western United States: A Scoping Review and Narrative Synthesis

S2.1. Protocol Elements

Element	Specification
Design	Scoping review with systematic searches and narrative synthesis.
Framework	Population-concept-context approach; reporting using PRISMA-ScR, with a PRISMA-ScR-style flow diagram.
Population/context	Rural, remote, frontier, Indigenous, underserved, western Canadian, and western/frontier U.S. communities; mechanistically transferable rural Canadian/U.S. studies retained where appropriate.
Concept	Telehealth, virtual care, eConsult, provider-to-provider telehealth, remote monitoring, telementoring, tele-emergency, and hybrid virtual/in-person care.
Primary outcomes	Timeliness, travel avoided, specialist input, attachment, ED/urgent-care use, continuity, clinician support, patient experience, safety, equity, cultural safety.
Synthesis domains	Infrastructure; patient-facing care; hybrid primary care; provider-to-provider/eConsult/telementoring; emergency/specialty/chronic/mental health; equity and cultural safety.
Transferability rule	Evidence outside western Canada/western U.S. retained only when the rural access mechanism was applicable and limitations were explicitly noted.
Protocol availability	Structured protocol supplied here; not registered in a public registry.

S2.2. Full Search Strings

Source	Search string
PubMed/MEDLINE	(telehealth[tiab] OR telemedicine[tiab] OR "virtual care"[tiab] OR "remote consultation"[tiab] OR eConsult[tiab] OR "remote monitoring"[tiab] OR "provider-to-provider"[tiab] OR "Project ECHO"[tiab]) AND (rural[tiab] OR remote[tiab] OR frontier[tiab] OR northern[tiab] OR Indigenous[tiab] OR underserved[tiab]) AND (Canada[tiab] OR Alberta[tiab] OR "British Columbia"[tiab] OR Saskatchewan[tiab] OR Manitoba[tiab] OR Yukon[tiab] OR "Northwest Territories"[tiab] OR "United States"[tiab] OR Wyoming[tiab] OR Montana[tiab] OR Idaho[tiab] OR Nevada[tiab] OR Utah[tiab] OR Arizona[tiab] OR "New Mexico"[tiab] OR Colorado[tiab] OR Washington[tiab] OR Oregon[tiab] OR California[tiab]) AND (access[tiab] OR timeliness[tiab] OR travel[tiab] OR "primary care"[tiab] OR specialist[tiab] OR "emergency department"[tiab] OR continuity[tiab] OR equity[tiab] OR broadband[tiab]) AND ("2016/01/01"[Date - Publication] : "2026/05/21"[Date - Publication]).
Embase	('telehealth'/exp OR 'telemedicine'/exp OR telehealth:ti,ab OR telemedicine:ti,ab OR 'virtual care':ti,ab OR 'remote consultation':ti,ab OR econsult:ti,ab OR 'remote monitoring':ti,ab OR 'provider-to-provider':ti,ab OR 'project echo':ti,ab) AND ('rural health'/exp OR rural:ti,ab OR remote:ti,ab OR frontier:ti,ab OR northern:ti,ab OR indigenous:ti,ab OR underserved:ti,ab) AND (Canada:ti,ab OR Alberta:ti,ab OR 'British Columbia':ti,ab OR Saskatchewan:ti,ab OR Manitoba:ti,ab OR Yukon:ti,ab OR 'United States':ti,ab OR Wyoming:ti,ab OR Montana:ti,ab OR Idaho:ti,ab OR Nevada:ti,ab OR Utah:ti,ab OR Arizona:ti,ab OR Colorado:ti,ab OR Washington:ti,ab OR Oregon:ti,ab OR California:ti,ab) AND (access:ti,ab OR timeliness:ti,ab OR travel:ti,ab OR 'primary care':ti,ab OR specialist:ti,ab OR 'emergency department':ti,ab OR continuity:ti,ab OR equity:ti,ab OR broadband:ti,ab) AND [2016-2026]/py AND [humans]/lim.
CINAHL	(MH "Telehealth+" OR MH "Telemedicine+" OR telehealth OR telemedicine OR "virtual care" OR "remote consultation" OR eConsult OR "remote monitoring" OR "Project ECHO") AND (MH "Rural Health Services+" OR rural OR remote OR frontier OR northern OR Indigenous OR underserved) AND (Canada OR Alberta OR "British Columbia" OR Saskatchewan OR Manitoba OR Yukon OR "United States" OR Wyoming OR Montana OR Idaho OR Nevada OR Utah OR Arizona OR Colorado OR Washington OR Oregon OR California) AND (access OR timeliness OR travel OR "primary care" OR specialist OR "emergency department" OR continuity OR equity OR broadband), limited to English and 2016-2026.
Scopus	TITLE-ABS-KEY((telehealth OR telemedicine OR "virtual care" OR "remote

	consultation" OR eConsult OR "remote monitoring" OR "provider-to-provider" OR "Project ECHO") AND (rural OR remote OR frontier OR northern OR Indigenous OR underserved) AND (Canada OR Alberta OR "British Columbia" OR Saskatchewan OR Manitoba OR Yukon OR "United States" OR Wyoming OR Montana OR Idaho OR Nevada OR Utah OR Arizona OR "New Mexico" OR Colorado OR Washington OR Oregon OR California) AND (access OR timeliness OR travel OR "primary care" OR specialist OR "emergency department" OR continuity OR equity OR broadband)) AND PUBYEAR > 2015 AND PUBYEAR < 2027.
Cochrane Library	(telehealth OR telemedicine OR "virtual care" OR eConsult OR "remote monitoring" OR "Project ECHO") AND (rural OR remote OR frontier OR Indigenous OR underserved) AND (access OR primary care OR specialist OR emergency OR continuity OR equity), limited to 2016-2026 where interface filters permitted.
Google Scholar/publisher platforms	Targeted combinations of: rural telehealth western Canada; rural telehealth western United States; British Columbia Real-Time Virtual Support; Wyoming Medicaid telehealth usability; rural eConsult Canada; rural hybrid virtual primary care; Indigenous virtual care cultural safety; telehealth broadband rural health. Results were screened for relevance and de-duplicated against database records.
Official sources	Targeted searches of HRSA, AHRQ, Rural Health Information Hub, ISED Canada, CRTC, Statistics Canada, and relevant provincial/federal health-system pages for shortage-area, broadband, and virtual-care context. Official sources were not treated as intervention-effect evidence unless they reported original data.

S2.3. Data-Charting Form

Field	Definition
Bibliographic details	Author, year, title, journal/report source.
Design	Review, randomized/quasi-experimental, cohort/cross-sectional, qualitative, mixed-methods, implementation study, official context source.
Sample size	Number of participants, cases, clinicians, studies, or service records, as applicable.
Setting/rurality	Country, province/state, rurality definition, Indigenous/Tribal or frontier context where reported.
Telehealth model	Patient-facing virtual care, telephone/video, eConsult, provider-to-provider, remote monitoring, telementoring, tele-emergency, hybrid care.
Comparator/context	Usual care, pre-post period, rural/urban, virtual/nonvirtual, implementation context.
Access outcomes	Timeliness, travel, specialist access, attachment, ED/urgent care, continuity, patient experience.
Safety/equity outcomes	Diagnostic safety, escalation, medication safety, cultural safety, broadband/device/literacy barriers.
Key findings	Quantitative estimates or qualitative themes as reported by source.
Applicability	Direct western Canada/western U.S. evidence or mechanistically transferable evidence.
Limitations/appraisal	CASP-informed design limitations, indirectness, reporting concerns.

S2.4. Study-Level Critical Appraisal Matrix

Reference numbers in square brackets correspond to the reference list in the main manuscript

Source	Design appraisal	Main risk-of-bias/quality concerns	Indirectness/applicability	Overall judgment
Chu et al., 2021 [22]	Large administrative repeated cross-sectional study with clear rurality definition.	Pandemic-era confounding; billing-policy changes; utilization rather than outcomes.	Ontario evidence; mechanistically transferable to western Canada.	Moderate quality; moderate indirectness.
Butzner and Cuffee, 2021 [8]	Narrative review with transparent PubMed search window.	Narrative synthesis; limited formal appraisal; heterogeneous studies.	U.S. rural evidence broadly relevant; not western-specific.	Low to moderate quality.
Totten et al., 2024/AHRQ 2022 [9,10]	Comprehensive systematic review with dual review and strength-of-evidence assessment.	Many included studies observational/small; variable outcomes.	Highly relevant to rural provider-to-provider support; not limited to western U.S.	High review quality; domain certainty varies.
Jong et al., 2019 [23]	Relevant northern/rural telehealth implementation evidence.	Program-specific and limited generalizability.	Directly relevant to remote northern Canadian access mechanisms.	Moderate applicability; low/moderate certainty.
Burton et al., 2022 [24]	Qualitative study with clear methods and rural BC context.	Small sample; single micropractice; selection bias possible.	Direct western Canadian evidence.	Useful qualitative evidence; moderate confidence for implementation themes.
Buyting et al., 2022 [25]	Scoping review with relevant rural cardiovascular focus.	Heterogeneous interventions; limited hard outcomes.	Rural Canada; transferable to western chronic-care follow-up.	Moderate relevance; low/moderate certainty.
Lai et al., 2026 [26]	Scoping review of rural home-based digital health.	New evidence base; heterogeneity; limited effectiveness estimates.	Rural Canada; applicable to western rural implementation.	Moderate relevance; low/moderate certainty.
Fitzsimon et al., 2023 (BMC) [27]	Mixed-methods with physician survey and focus groups.	Small clinician sample; local program; self-report.	Ontario; mechanistically transferable to hybrid rural models.	Moderate relevance; low/moderate certainty.
Fitzsimon et al., 2023 (BMJ Open) [28]	Population-based comparative service evaluation.	Nonrandomized; pandemic-era trends; residual confounding.	Ontario; transferability requires local backup capacity.	Moderate quality; low certainty for causal ED effects.
Peixoto et al., 2024 [29]	Program evaluation of new patient	Nonrandomized, single-program	Rural Ontario evidence;	Moderate relevance; low certainty.

	attachment within a rural integrated virtual-care model.	evaluation; limited comparator and follow-up information.	mechanistically transferable to western rural attachment gaps where local team infrastructure exists.	
St-Amant et al., 2025 [30]	Mixed-methods patient-experience study using an online survey and semi-structured interviews.	Self-selection and response bias; single rural county; predominantly self-reported outcomes.	Rural Ontario evidence; transferable to patient navigation, satisfaction, and escalation-pathway themes.	Moderate relevance; low-to-moderate confidence for patient-experience findings.
Buchanan et al., 2025 [31]	Cross-sectional patient survey.	Response bias; selected enrolled population.	Ontario; transferable to patient experience in hybrid attachment models.	Moderate relevance; low certainty.
Lauscher et al./Ho et al., Real-Time Virtual Support papers [32–34]	Descriptive case and health-system reports.	Limited controlled outcome data.	Direct western Canadian evidence, including First Nations/rural contexts.	High relevance; low/moderate certainty.
Harkey et al., 2020 [35]	Systematic review of rural rehabilitation telehealth satisfaction.	Only four included studies; limited clinical outcomes.	Not western-specific; satisfaction mechanism transferable.	Low/moderate quality.
Watanabe et al., 2023 [36]	Systematic review of six RCTs.	Small number of trials; heterogeneous diagnoses.	Rural mental health broadly relevant.	Moderate review quality; low/moderate domain certainty.
Homer et al., 2024 [37]	Survey of Wyoming Medicaid members.	Survey design; self-reported usability; limited clinical outcomes.	Direct western/frontier U.S. evidence.	High relevance; moderate risk of bias.
Moecke et al., 2024 [13]	Scoping review with Indigenous telehealth focus.	Mapping design; variable study quality.	Relevant to Indigenous and Tribal implementation across included countries.	High conceptual relevance; moderate confidence for cultural-safety themes.
Fitzpatrick et al./Roach et al., ARQS evidence [14–16]	Rapid review and qualitative Indigenous virtual-care studies.	Context-specific; qualitative/rapid review designs.	Highly relevant to western Canadian Indigenous virtual primary care.	High relevance; moderate confidence for implementation themes.
Liddy et al., eConsult studies [38,39]	Cross-sectional and utilization studies with large service datasets.	Observational; rural definitions and outcomes limited to service use.	Canadian; transferable to rural specialist access.	Moderate quality and relevance.
McBain et al./AHRQ Project ECHO [40,41]	Systematic review and official program context.	Provider outcomes often self-reported; patient-outcome evidence weaker.	Rural/underserved workforce support broadly transferable.	Moderate relevance; low/moderate certainty.
